# Transcript Abundance of *Photorhabdus* Insect-Related (Pir) Toxin in *Manduca sexta* and *Galleria mellonella* Infections

**DOI:** 10.3390/toxins8100287

**Published:** 2016-09-29

**Authors:** Anaïs Castagnola, Geraldine Mulley, Nathaniel Davis, Nicholas Waterfield, S. Patricia Stock

**Affiliations:** 1 Center for Insect Science, University of Arizona, Tucson, AZ 85721, USA; anais@email.arizona.edu; 2 School of Biological Sciences, University of Reading, Whiteknights, Reading RG6 6AJ, UK; g.mulley@reading.ac.uk; 3 Pima Community College, Tucson, AZ 85721, USA; ndavis11@mail.pima.edu; 4 Division of Biomedical Sciences, Warwick Medical School, Warwick University, Coventry CV4 7AL, UK; N.R.Waterfield@warwick.ac.uk; 5 Department of Entomology, University of Arizona, Tucson, AZ 85721, USA

**Keywords:** *Photorhabdus luminescenslaumondii*, pir toxin, *pirA* and *pirB* transcript detection, portal of entry, tissue specificity

## Abstract

In this study, we assessed pirAB toxin transcription in *Photorhabdus luminescens laumondii* (strain TT01) (Enterobacteriaceae) by comparing mRNA abundance under in vivo and in vitro conditions. In vivo assays considered both natural and forced infections with two lepidopteran hosts: *Galleria mellonella* and *Manduca sexta*. Three portals of entry were utilized for the forced infection assays: (a) integument; (b) the digestive route (via mouth and anus); and (c) the tracheal route (via spiracles). We also assessed *plu4093-2* transcription during the course of a natural infection; this is when the bacteria are delivered by *Heterorhabditis bacteriophora* nematodes. Transcript abundance in *G. mellonella* was higher than in *M. sexta* at two of the observed time points: 15 and 18 h. Expression of *pirAB plu4093-2* reached above endogenous control levels at 22 h in *G. mellonella* but not in *M. sexta*. Overall, *pirAB plu4093-2* transcripts were not as highly expressed in *M. sexta* as in *G. mellonella,* from 15 to 22 h. This is the first study to directly compare *pirAB plu4093-2* toxin transcript production considering different portals of entry.

## 1. Introduction

The entomopathogenic Gram-negative bacterium *Photorhabdus luminescens laumondii* (strain TT01) (Enterobacteriaceae) and its nematode host, *Heterorhabditis bacteriophora* (Nematoda: Heterorhabditidae), form an insecticidal partnership that has been shown to successfully kill a wide range of insect pests [1,2,3]. Third-stage infective juvenile (IJ) nematodes are the only free-living stage in the nematode’s life cycle and are responsible for vectoring *Photorhabdus* from one host to another [4,5]. Once a suitable insect host is found, IJs may penetrate it through three different routes: (a) integument; (b) the digestive route (i.e., IJs penetrate into the insect through either the mouth or anus and are readily exposed to gut tissue); and (c) the tracheal route (i.e., IJs enter through the insect’s spiracles) [6,7,8]. Although it has not yet been definitively demonstrated all the locations at which the nematodes can release bacteria during these different routes of infection, it has been shown to predominantly occur in the hemolymph. Nevertheless, anytime *Photorhabdus* cells are released by the IJs, they subsequently colonize insect tissue, and the anterior portion of the midgut is one of the initial targets. More specifically, the *Photorhabdus* cells align to the intestinal longitudinal muscles prior to invading the fat body tissue [9].

An examination of the genome of *P. luminescens laumondii* (here after *P. l. laumondii*), strain TT01, revealed the presence of a number of pathogenicity islands that contain a multitude of toxins and virulence factors that can contribute to insect mortality [10]. For example, the insect toxin complex proteins, Tc, have been shown to attack the anterior grooves of the intestine in *M. sexta* (Lepidoptera: Sphingidae) [11]. Another toxin, the Mcf (makes caterpillars floppy) protein has been shown to disrupt gut tissue and induce apoptosis in hemocytes, ultimately making caterpillars floppy due to a loss of turgor pressure and thus giving the toxin its unique name [12,13]. Additionally, the *Photorhabdus* virulence cassettes (PVCs), which have a phage-like appearance, have been found to kill hemocytes and cause cytoskeletal condensation [14]. Furthermore, the *Photorhabdus* insect-related (PirAB) toxins, which are encoded in two independent loci, *plu4437*-*plu4436* and *plu4093*-*plu4092*, each elaborate a distinct binary toxin [5]. The two PirABs have been shown to have variable toxicity depending on the delivery method and insect host. For example, protein products of *plu4093-2* and *plu4437-6* have oral toxicity against *Plutella xyllostella* and mosquito larvae [10,15]. In addition, cloned PirAB (*plu4093-2*) has injectable toxicity in *G. mellonella* but no oral or injectable toxicity in *M.* *sexta,* nor oral activity in *H. virescens* or *L. dispar* [16]. PirAB (*plu4437-6*) showed no oral or hemocoel-based insecticidal activity to *G. mellonella* or *S. litura* [17]. Interestingly, the PirAB toxin from *P. asymbiotica* has been shown to exhibit oral toxicity against the larvae of the Dengue vector mosquitos *Aedes aegypti* and *Aedes albopictus* but did not affect the *Mesocyclops thermocyclopoides* predator [18]. Although purified PirA and PirB are toxic when applied together, the linking of the two proteins, using artificial translation fusion, has been shown to have a cytotoxic effect in vitro against cultured CF-203 midgut cells of the spruce budworm, *Choristoneura fumiferana*. These artificial translational fusions did not show toxicity when orally delivered in *S. exigua* larvae but had injectable toxicity in *S. exigua* [19]. Recently, a plasmid containing a gene homologous of Pir toxins was found in the shrimp pathogen *Vibrio parahaemolyticus* [20,21]. The affected shrimp show pathological responses suggesting the involvement of Pir toxin-like proteins in the etiology of acute hepato-pancreatic necrosis disease [20].

Pir proteins have amino acid similarity with the pore-forming domain of Cry2A toxin of *Bacillus thuringiensis* (Bt) [22]. This sequence correspondence has prompted the development of toxin chimeras with the goal of enhancing their individual toxicity [23]. For example, when PirB was fused with Cry2A, the recombinant toxin displayed oral toxicity in *S. exigua* larvae, but no oral toxicity was observed in a different host (*Helicoverpa armigera*), suggesting this chimera is host specific [23]. 

Although the toxicity and relationship to virulence of PirAB have both been investigated, the expression of this toxin during the natural course of infection has yet not been assessed. Therefore, in this study, we evaluated the abundance of *pirAB* toxin transcripts of the *Photorhabdus l. laumondii* TT01 strain when delivered by its vector nematode, *H. bacteriophora.* Natural and forced infection assays were also investigated.

## 2. Results

### 2.1. PirAB plu4093-2 Transcript Expression in Natural Infections

**Figure 1:**
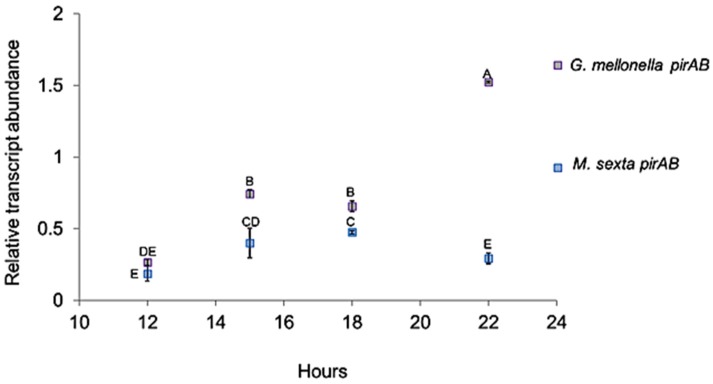
*PirAB* transcript abundance detected by qPCR during the course of natural infection in *G. mellonella* and *M. sexta*. Different letters denote statistically significant means.

The relative abundance of the pirAB transcript was assessed for both lepidopteran hosts at four time points: 12, 15, 18, and 22 h, considering natural infections (i.e., larvae were exposed to infective juvenile nematodes (IJs)). Our results show that transcript expression in both *G. mellonella* and *M. sexta* remained below the endogenous control between 12 and 18 h post-IJ exposure. Transcript abundance in *G. mellonella* was slightly higher (Student’s *t*-test α = 0.05) than in *M. sexta* at three time points: 12, 15, and 18 h (Figure 1). However, a three-fold increase (Students *t*-test α = 0.05) of pirAB transcripts was detected at 22 h in *G. mellonella* when compared with that observed in *M. sexta.*

### 2.2. Portal of Entry and Time Until Death in Forced Infections

Time until death in *G. mellonella* ranged between 21 and 24 h across different portals of entry (POEs) (Table 1). This timing can be correlated with the increase in *pirAB* toxin transcript production that was observed in the natural iinfection assays (Figure 1). Our data also suggest that, except for the anus, all other portals of entry to a host had no significant effect on time to death.

Time until death in *M. sexta,* compared with *G. mellonella,* was delayed, varying between 25 to 33 h (Table 1). The longest time to death was observed when IJs were delivered via the mouth (Table 1) and is statistically longer when compared with both spiracle and tegument IJ injections. Time until death by both the digestive tract entry routes (mouth and anus) were not significantly different from each other.

### 2.3. Transcript Abundance in Forced Infection Assays

*PirAB* *plu4093-2* transcript abundance was measured considering different POEs at two observation time points: 20 min and 2 h post-injection for the two insect hosts tested.

**Figure 2:**
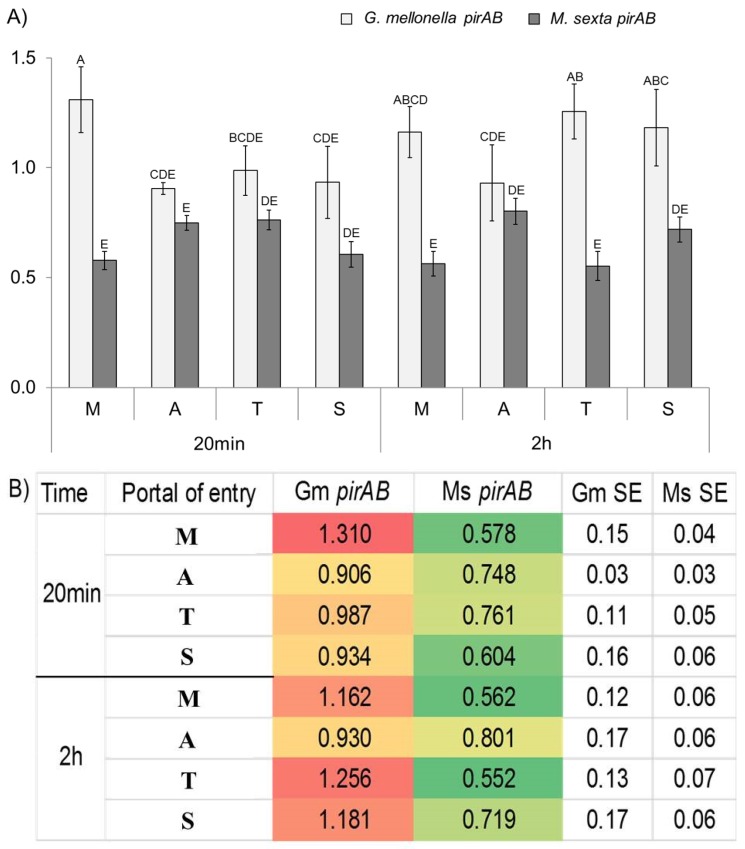
(**A**) Bar graph of *pirAB* transcripts in forced injection assays considering different portals of entry (M: Mouth, A: Anus, T: Tegument, S: Spiracle). Different letters denote statistically significant means. (**B**) Heat map of *pirAB* transcript abundance detected by qPCR using relative standard curve method. Gm = *G. mellonella*, Ms = *M*. *sexta.*

For *G. mellonella*, the highest expression of *pirAB* transcripts was observed in the mouth at 20 min post-injection. Expression of this toxin in all other POEs was significantly lower (Figure 2). At 2 h post-infection, toxin expression was moderately expressed in mouth, tegument, and spiracles and remained low for the anus. 

Contrastingly, in *M. sexta*, *pirAB* transcripts were not as highly expressed as in *G. mellonella*. *PirAB* transcript expression at all portals of entry was about the same. Anus and tegument routes were minimally higher at 20 min post-infection; however, differences were not significant (*p* < 0.05). Transcript expression for the mouth and spiracles entries showed a low expression profile at this time interval. At 2 h post-injection, transcription profiles remained similar to those recorded at 20 min (Figure 2). 

## 3. Discussion

*Photorhabdus luminescens* PirAB toxins have been reported to possess both injectable and oral activities against a range of insects [10,15,16,17]. It has also been demonstrated that their toxicity is binary and host-specific [15,16,17,22]. For example, histological examination of *P. xylostella* larvae fed with *E. coli* expressing PirA/PirB proteins revealed midgut epithelium abnormalities, suggesting gut toxicity [15]. Furthermore, PirAB toxins have shown oral activity against mosquito larvae [10,18]. There is also evidence that, in *G. mellonella*, both PirA and PirB components, from both *plu4093-2* and *4437-6*, are necessary for injectable activity because injection of either PirA or PirB alone does not cause mortality [16,17]. Blackburn et al. also showed that, when the *plu4092* gene was truncated, mortality of *P. xylostella* decreased from 100% to <15% [15]. In all these studies, the approach for assessing activity of Pir toxins has considered the injection of bacteria cells or toxin preparations delivered into hemocoel via tegument or orally via the ingestion of PirAB toxin. Additionally, Pir-Cry toxin chimera delivery was through oral delivery by the incorporation of toxin combinations into the insect diet [23]. Until now, the expression of Pir toxins when the *Photorhabdus* are delivered by their vectoring nematodes had not been investigated.

In this study, we evaluated abundance of *pirAB* toxin transcripts (*plu4093-2 and plu4437*) of the *P. l. laumondii* TT01 strain. Furthermore, until now, expression of Pir toxins when the bacteria are naturally delivered by their vector nematodes had not been investigated. In this respect, we investigated the transcription of independent *pirAB* loci, *plu4093-2* and *plu4437-6,* in insect homogenates and under in vitro conditions (Suppl. Materials). Our observations showed that the addition of *M. sexta* hemolymph to LB broth slightly reduced the transcription of all the *pir* genes. Furthermore, there was a higher decrease of *plu4093-2* than *plu4437-6.* However, the overall mean base mapping value for *plu4093-2* was higher than *plu4437-6* (Tables S1 and S2, Figure S1). We also observed that all *pir* genes were more highly expressed during the exponential growth phase other than the lone *pirA*. There was a big increase in expression of the *pirA* orphan gene at the stationary phase, while the other *pir* genes showed only minor differences. Based on these results, we only focused on expression of *plu4093-2* for the subsequent experiments*.*

Our results showed that *plu4093-2* *pirAB* transcripts were transcribed as early as 22 h during the course of a natural infection. However, transcript levels remained below threshold levels between 12 h and 20 h post-infection. Interestingly, a three-fold increase of transcripts was detected at 22 h post-infection in *G. mellonella* but not in *M. sexta*. In this respect, we speculate that this may be due to the fact that *M. sexta* is a more resilient host to *Photorhabdus–Heterorhabditis* infection. Therefore, the observed low expression of *pir* transcripts in *M. sexta* may be due to the fact that more time was needed to show their activity.

With respect to forced infection assays, our data showed that, for *G. mellonella*, *pir* toxin transcripts are highly expressed in the mouth at both 20 min and 2 h post-injection in *H. bacteriophora* IJs. We speculate that IJ nematodes may be able to release *Photorhabdus* cells when they enter a host through the mouth. Alternatively, it is possible that IJs may be crushed by the insect’s mouth parts, thereby allowing the ectopic release of bacterial cells and thus activation of gene expression. 

Other studies have also demonstrated that activation of toxin gene expression of bacteria responding to insect tissue signals [24,25,26]. Specifically, it has been shown that exposure of *P. luminescens* cells to *G. mellonella* tissue homogenates activates production Tc and other putatively identified toxins [24]. In contrast, toxin expression in *M. sexta* was low for all portals of entry at both time points tested (Figure 2). In this respect, we speculate that *M. sexta* may have a more efficient immune system than *G. mellonella*; therefore, additional time may be required to propel toxin production and cause insect death. In relation to this, studies have shown that *M. sexta* can mount a defense against *P. luminescens* [25]. Low toxin transcript measurements reported in this study are consistent with observations by Daborn et al., who demonstrated that *Photorhabdus tc* toxin gene expression could not be detected above threshold levels at early infection time points [26]. 

Overall, this study demonstrated for the first time the transcription of *pirAB* in natural infections when *P. l. laumondii* cells are delivered by *Heterorhabditis* IJs. We also showed a three-fold increase of PirAB toxin transcription at 22 h in *G. mellonella*, suggesting the deployment of toxin transcripts in early stages of the infection process. Further work is needed to discern the timing of *plu4093-2* and *plu4437-6* translation and potential for component PirA and PirB complex formation. Furthermore, results from *G. mellonella* forced injection assays correlated time until death with *pirAB* toxin transcript production. Additionally, we showed that *pirAB* transcripts are produced in *G. mellonella* larvae when the bacteria are delivered by *H. bacteriophora* IJs via the mouth. 

## 4. Experimental Section

### 4.1. Insects, Nematodes, and Bacterial Cultures

Fifth-instar larvae of the greater wax moth, *Galleria mellonella* (Lepidoptera: Pyrallidae), and early fourth instar *M. sexta* (Lepidoptera: Sphingidae) larvae were considered for the natural and forced infection assays. Selection of hosts was based on their known susceptibility to *Heterorhabditis*–*Photorhabdus* infection [27]. *G. mellonella* larvae were obtained from Timberline Fisheries (Timberline, Marion, IL, USA) and kept in the lab at 15 °C until their use in the experiments. *M. sexta* larvae were obtained from *J. Hildebrandt’s* laboratory at the University of Arizona. Prior to the experiments, larvae were surface sterilized with 70% ethanol. 

*Heterorhabditis bacteriophora* strain TT01 was obtained from *I. eleftherianos* laboratory (G. Washington University, Washington, DC, USA). Nematodes were propagated in *G. mellonella* larvae following procedures described by Stock and Goodrich-Blair (2012) [28]. IJs were collected from modified White traps and stored in 250-mL tissue culture flasks (at a concentration of 15,000 IJs/mL) at 15 °C until their use in the experiments following procedures described by Stock and Goodrich-Blair (2012) [28].

### 4.2. Natural and Forced Infection Assays

Two different experimental arenas and inocula were considered for the natural infection and forced injection assays depending on the insect host tested. For the natural infection assays, a 1.7-mL micro-centrifuge tube lined with a small triangle of filter paper (Whatman #1) was used for *G. mellonella* infections. An inoculum of 110 IJs/larva was delivered on the filter paper, and one larva was added to each tube. A 15-mL conical centrifuge tube was considered for *M. sexta* infections. An inoculum of 500 IJs was delivered on a small triangle of filter paper (Whatman #1) placed inside the tube.

**Figure 3:**
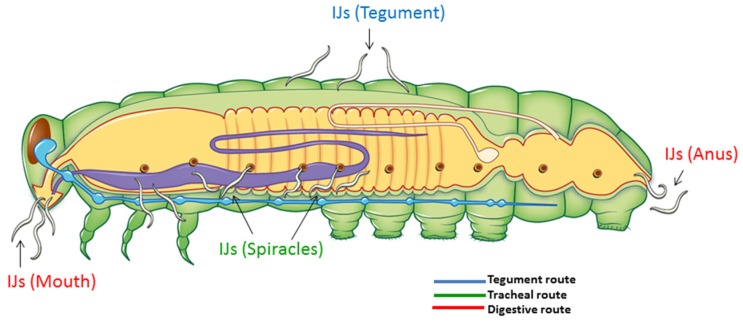
Schematic representation showing portals of entry considered and tissue targets for the forced infection assays.

Forced injections were administered by delivering the same concentration of *H. bacteriophora* IJs into *G. mellonella* or *M. sexta* considering four portals of entry (POE): mouth, anus, spiracles, and cuticle (Figure 3). For this purpose, a single larva was injected with a syringe with a 25-gauge needle considering one portal of entry at a time and controls consisted of larvae injected with M9 buffer solution only. In both cases, one larva was added into each tube. To allow air exchange, the lid of each tube was perforated. Tubes were capped and placed in an incubator in the dark and maintained at 28 °C. There were six tubes per observation time points: 12 h, 15 h, 18 h, and 22 h for natural infections or 20 min and 2 h for forced injections. RNA from *H. bacteriophora* IJs was collected at these time points. Three replicates were collected per time point, and experiments were repeated twice. 

A remote recording video system was built using iSpy Connect Open Source Camera Security Software (iSPY Group, Stockholm, Sweden) and a mounted webcam to record the course of infection for the forced infection assays. A 24-well plate was used as the experimental arena, and one larva per well was added. Three replicates were collected per time point and experiments were repeated twice. A time-lapse video was created with Windows MovieMaker software 2012 (Microsoft Corp., Redmond, WA, USA) to measure time stamp information to record time until death to establish mean time until death. 

### 4.3. Detection of pirAB Transcripts through qPCR

Total RNA was extracted using TriReagent (Molecular Research Center, Cincinnati, OH, USA) according to the manufacturer’s instructions. Contaminating genomic DNA was eliminated by digestion with DNAse I (GE Healthcare, Piscataway, NJ, USA) for one hour at 37 °C. Samples were further purified using the RNA clean-up and concentration kit (Thermo Fisher Scientific, Hampton, NH, USA). Purified total RNA was checked for quality and quantified by 2% agarose gel electrophoresis and Nanodrop spectrophotometer (Nanodrop Technologies, Wilmington, DE, USA). 

One microgram of total RNA, which did not amplify from PCR with endogenous control (primer pair 5 and 6 with DreamTaq K1071, according to Thermo’s recommendations) (Table 2), was considered gDNA-free and then used to synthesize cDNA. Reactions were designed according to the RevertAid First Strand cDNA Synthesis Kit (Thermo Scientific, Waltham, MA, USA) according to the manufacturer’s instructions. Relative quantification of *pirAB* gene expression was measured in a Mastercycler^®^ ep gradient S (Eppendorf, San Diego, CA, USA) for forty cycles using the Realplex^4^ version 2.2 software (Eppendorf, San Diego, CA, USA) to collect cycle threshold (*C*_t_) values. All primers used in qRT-PCR are presented in Table 2.

Primers targeting *pirAB* transcripts were designed using Geneious software version 8.0.5 (Biomatters, Auckland, New Zealand) and NCBI primer-blast. For relative quantification, an endogenous control, Gyrase subunit B (*gyrB*) (*P. l. laumondii* TTO1 *plu004*), was considered. The relative standard curve method was used for gene expression analysis comparing transcript levels between target and endogenous control genes between treatments. Primers (Table 2) were validated by a clean melting curve and Sanger sequencing analysis. 

### 4.4. Statistical Analysis

Statistical analysis was performed using JMP^®^ software version 12.2.0 Copyright^©^ SAS Institute Inc. where a comparison of means was performed using Student’s *t*-test at an alpha of 0.05. For all figures and tables, unique letters denote statistical significance. 

## Figures and Tables

**Table 1:** Time until death based on portal of entry in *G. mellonella* and *M. sexta.*

Portal of Entry	Time Until Death (h ± SE)	
	G. mellonella *	M. sexta *
Mouth	21 ^a^ ± 0.72	33 ^a^ ± 2.47	
Anus	24 ^b^ ± 0.85	28 ^a,b^ ± 2.31	
Spiracle	23 ^a,b^ ± 1.21	26 ^b^ ± 1.42	
Tegument	22 ^a,b^ ± 0.74	25 ^b^ ± 1.65	

* Unique letters for each species column denote statistical significance based on means comparison using Student’s *t*-test (*p* < 0.05). Different letters indicate statistically significant differences.

**Table 2:** Primers used in this study.

Toxin Transcript Detected	Gene ID	Amplicon Size (bp)	Sequence	Tm (°C)	GC (%)
*pirAB*	*plu4093-2*	231	GGCACGTTAACACCTTCTCT	58	48
			TGCAGTTGGTCCTTTGAGTGA	62	48
*gyrB*	*plu004*	105	CTCGTGAAGCAGCCCGTAAA	64	55
			CCGGATCACGTTCCTGACAA	64	55

## Supplementary Materials

The following are available online at www.mdpi.com/2072-6651/8/10/287/s1., Figure S1: *Pir*-loci expression in *P. luminescens* TT01 strain grown under two different media, Table S1: DESeq analysis of RNA-seq data showing the mean mapped base counts (across the ORFs) and log2 fold change for each of the *pir* gene homologues in *P. luminescens* TT01 grown in different media, LB alone or with 20% *v*/*v* *M. sexta* hemolymph supplement (LB+MSH). Different *pir*-operons are colour coded. E and S represent mid-exponential and stationary growth phases respectively, Table S2: DESeq comparison of exponential and stationary phase RNA-seq data showing mean mapped base counts (across the ORFs) and log2 fold change for each of the *pir* gene homologues in *P. l. laumondii* TT01 grown in the different media, LB alone or with 20% *v*/*v* *M. sexta* hemolymph supplement (LB+MSH). The three different *pir*-operons are colour coded.
