# Supplementary material for: Transcript Abundance of Photorhabdus Insect-Related (Pir) Toxin in Manduca sexta and Galleria mellonella Infections
Source: Toxins (Basel). 2016 Sep 29;8(10):287. doi: 10.3390/toxins8100287 (PMC5086647; doi:10.3390/toxins8100287)
Supplement: Supplementary file 1 [file toxins-08-00287-s001.pdf]

Supplementary Materials: Transcript Abundance of *Photorhabdus* Insect Related (Pir) Toxin in *Manduca sexta* and *Galleria mellonella* Infections

Anaïs Castagnola, Geraldine Mulley, Nathaniel Davis, Nicholas Waterfield and S. Patricia Stock

In Vitro Assays

To complement qRT-PCR studies we also performed one single run pilot RNA-seq analysis of *P. l. laumondii* grown in LB medium at different growth phases and in the presence or absence of cell-free *M. sexta* hemolymph supplements. It should be noted that this data was intended for comparative purposes only. These RNA-seq experiments had no biological replicates. Specifically, detection of *pirAB* transcripts in vitro considering the following media: Lysogeny Broth (LB), and LB+ cell free insect hemolymph (20% *v*/*v*). The goal was to confirm if *plu4092* and *plu4437-6* were both produced during in vitro growth and to compare and/or illustrate results from the qRT-PCR experiments.

1. Experimental Design

1.1. RNA Purification

Cultures of *P. l. laumondii* TT01 were grown overnight in 10 mL LB at 28 °C, 250 rpm, and sub-cultured (1:100 dilution) in 50 mL media at 28 °C in a 250 mL flask for 4 h to mid-log phase (OD_{600} ~0.6). To extract total RNA, a 10 mL aliquot of each culture (~8 × 10^8 cells) was added to 25 mL RNA *later* (Ambion, ThermoFischer Scientific, Waltham, MA, USA) and centrifuged at 10,000 rpm, 4 °C in a JA-25.50 rotor centrifuge (Beckman Coulter Beckman, CA, USA). RNA was isolated from bacterial pellets using the miRNeasy kit (Qiagen, Hilden, Germany), with an on-column DNase treatment (Qiagen), and RNA eluted in 70 µL RNAse-free H_2O. To ensure complete removal of DNA, a subsequent DNase treatment was performed using the Turbo™ DNase-free kit (Ambion, ThermoFischer Scientific, Waltham, MA, USA). The concentration and integrity of RNA samples was determined with an Experion RNA StdSens analysis kit (Bio-Rad Laboratories, Hercules, CA, USA).

1.2. RNA-seq Library Preparation

Ribosomal RNA was depleted from RNA samples using the Ribo Zero kit (Takara, Mountain View, CA, USA) for Gram-negative bacteria. rRNA depletion was verified and samples quantified using a 2100 Bioanalyzer with a RNA 6000 Pico kit (Agilent, Santa Clara, CA, USA). Strand-specific RNA-seq libraries were constructed using the Illumina compatible ScriptSeq mRNA-seq library preparation kit (Epicentre®, Illumina, San Diego, CA, USA). cDNA libraries were quantified using a 2100 Bioanalyzer with a DNA 1000 kit (Agilent, Santa Clara, CA, USA). Each library was sequenced on the HiSeq 2000 (Illumina,), multiplexed with10 libraries/lane, with 100 bp paired-end reads by Source Bioscience (UK).

1.3. RNA-seq Analysis

RNA-seq data analysis was performed on Linux servers running debian OS, with 96 GB RAM. A comparative analysis was performed between treatments. For each treatment, the raw data in fastq format was converted to bfq format. The MAQ alignment software (version 0.7.1, Mountain View City, CA, USA) [1] was used to align the Illumina data to the *P. l. laumondii* TT01 genome obtained from Genbank. Custom PERL scripts were then used to count the number of reads aligned to each gene and convert the data into a format suitable for statistical analysis.

The data was normalised by reads per kilobase of exon per million mapped reads (RPKM). The RPKM measure was chosen as read density reflects the molar concentration of a transcript in the starting sample by normalizing for RNA length and for the total number of reads in the measurement.

RPKM normalization enables comparison of transcript levels both within and between samples [2]. Treatments were then compared using DESeq, an R package that estimates variance-mean dependence in count data derived from RNA-seq experiments and tests for differential expression based on a model using the negative binomial distribution, to identify differentially expressed genes from different samples [3]. Using DESeq it was possible to generate text files containing the expression values for the samples, and a *p*-value for each gene to denote its expression difference between libraries. In addition the RNA-seq data was visualised using the methods described by Croucher et al [4]. Reads were aligned to the genome of *P. luminescens* TT01 using SSAHA2 (version1.0.9, Sanger Institute, Hinxton, UK) [5]. The cigar2Coverage PERL script was used to convert the SSAHA2 output into a format compatible with the Artemis genome browser. This allowed the mapped transcriptome data to be viewed, in a strand-specific manner, as a graph relative to the genome annotation.

2. Results

Tables S1 and S2 show DESeq comparisons of *pirAB* gene expression in *P. l. laumondi* at different growth phases in LB media in the presence and absence of *M. sexta* blood supplements (20% *v*/*v*). Abundance of locus 4093-2 was two times higher than locus 4437-6. Abundance of locus 4437-5 showed a higher fold change in the presence of insect blood compared to locus 4093-2, 0.07 and 0.41 compared to −0.67 and −0.7, respectively (Figure S1).

Table S1. DESeq analysis of RNA-seq data showing the mean mapped base counts (across the ORFs) and $\log_2$ fold change for each of the *pir* gene homologues in *P. luminescens* TT01 grown in different media, LB alone or with 20% *v*/*v M. sexta* hemolymph supplement (LB+MSH). Different *pir*-operons are colour coded. E and S represent mid-exponential and stationary growth phases respectively.

Gene	Protein	LB Base Mean	LB+MSH Base Mean	$\log_2$ Fold Change
plu2979	PirA orphan	743	220	−1.76, E
		2279	1429	−0.19, S
plu4092	PirB	707	435	−0.67, E
		1002	1054	−0.99, S
plu4093	PirA	57	77	−0.7, E
		19,030	16,725	−2.51, S
plu4436	PirB	2043	1028	0.07, E
		530	92	0.44, S
plu4437	PirA	170	231	0.41, E
		14	42	1.58, S

Table S2. DESeq comparison of exponential and stationary phase RNA-seq data showing mean mapped base counts (across the ORFs) and log2 fold change for each of the *pir* gene homologues in *P. l. laumondii* TT01 grown in the different media, LB alone or with 20% *v/v M. sexta* hemolymph supplement (LB+MSH). The three different *pir*-operons are colour coded.

Culture conditions	Gene	Protein	Exponential Base Mean	Stationary Base Mean	Log2 Fold Change
LB+MSH	*plu2979*	PirA orphan	136	23,806	−7.44
LB+MSH	*plu4092*	PirB	888	1463	−0.72
LB+MSH	*plu4093*	PirA	270	131	1.03
LB+MSH	*plu4436*	PirB	655	329	0.99
LB+MSH	*plu4437*	PirA	47	60	−0.34
LB	*plu2979*	PirA orphan	30,557	523	−5.87
LB	*plu4092*	PirA	3281	1604	−1.03
LB	*plu4093*	PirB	851	497	−0.77
LB	*plu4436*	PirA	273	705	1.36
LB	*plu4437*	PirB	22	40	0.83

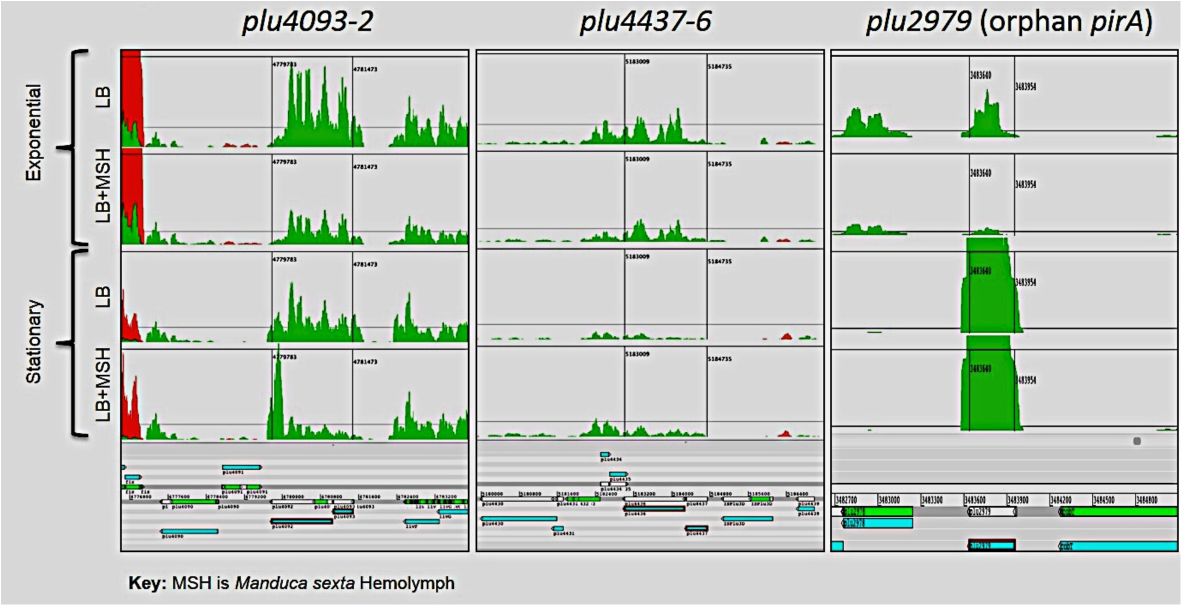

Figure S1. *Pir*-loci expression in *P. luminescens* TT01 strain grown under two different media.

References

1. Mapping and Assembly with Quality. Available online: http://sourceforge.net/projects/maq/ (accessed on 29 September 2016)).
2. Mortazavi, A.; Williams, B.A.; McCue, K.; Schaeffer, L.; Wold, B. Mapping and quantifying mammalian transcriptomes by RNA-Seq. *Nat. Methods* **2008**, *5*, 621–628.
3. Anders, S.; Huber, W. Differential expression analysis for sequence count data. *Genome Biol.* **2010**, *11*, doi:10.1186/gb-2010-11-10-r106.
4. Croucher, N.J.; Fookes, M.C.; Perkins, T.T.; Turner, D.J.; Marguerat, S.B.; Keane, T.; Quail, M.A.; He, M.; Assefa, S.; Bahler, J.; et al. A simple method for directional transcriptome sequencing using Illumina technology. *Nucleic Acids Res.* **2009**, *37*, doi:10.1093/nar/gkp811.
5. Ning, Z.; Cox, A.J.; Mullikin, J.C. SSAHA: A fast search method for large DNA databases. *Genome Res.* **2001**, *11*, 1725–1729.
